# Safety and audiological outcome in a case series of tertiary therapy of sudden hearing loss with a biodegradable drug delivery implant for controlled release of dexamethasone to the inner ear

**DOI:** 10.3389/fnins.2022.892777

**Published:** 2022-09-20

**Authors:** Stefan K. Plontke, Arne Liebau, Eric Lehner, Daniel Bethmann, Karsten Mäder, Torsten Rahne

**Affiliations:** ^1^Department of Otorhinolaryngology, Head and Neck Surgery, Martin Luther University Halle-Wittenberg, Halle, Germany; ^2^Institute of Pathology, Martin Luther University Halle-Wittenberg, Halle, Germany; ^3^Institute of Pharmacy, Martin Luther University Halle-Wittenberg, Halle, Germany

**Keywords:** glucocorticoids, intracochlear, biodegradable polymer, intratympanic, human, salvage therapy, round window, scala tympani

## Abstract

**Background:**

Intratympanic injections of glucocorticoids have become increasingly common in the treatment of idiopathic sudden sensorineural hearing loss (ISSHL). However, due to their fast elimination, sustained applications have been suggested for local drug delivery to the inner ear.

**Materials and methods:**

The study is based on a retrospective chart review of patients treated for ISSHL at a single tertiary (university) referral center. We included patients who were treated with a solid, biodegradable, poly(D,L-lactic-co-glycolic acid) (PLGA)-based drug delivery system providing sustained delivery of dexamethasone extracochlear into the round window niche (*n* = 15) or intracochlear into scala tympani (*n* = 2) for tertiary therapy of ISSHL in patients without serviceable hearing after primary systemic and secondary intratympanic glucocorticoid therapy. We evaluated the feasibility and safety through clinical evaluation, histological examination, and functional tests [pure-tone threshold (PTA), word recognition scores (WRS)].

**Results:**

With adequate surgical preparation of the round window niche, implantation was feasible in all patients. Histologic examination of the material in the round window niche showed signs of resorption without relevant inflammation or foreign body reaction to the implant. In patients where the basal part of scala tympani was assessable during later cochlear implantation, no pathological findings were found. In the patients with extracochlear application, average preoperative PTA was 84.7 dB HL (SD: 20.0) and 76.7 dB HL (SD: 16.7) at follow-up (*p* = 0.08). The preoperative average maximum WRS was 14.6% (SD: 17.9) and 39.3% (SD: 30.7) at follow-up (*p* = 0.11). Six patients (40%), however, reached serviceable hearing. The two patients with intracochlear application did not improve.

**Conclusion:**

The extracochlear application of the controlled release system in the round window niche and – based on limited observations - intracochlear implantation into scala tympani appears feasible and safe. Due to the uncontrolled study design, conclusions about the efficacy of the treatment are limited. These observations, however, may encourage the initiation of prospective controlled studies using biodegradable controlled release implants as drug delivery systems for the treatment of inner ear diseases.

## Introduction

There is no approved medical therapy for idiopathic sudden sensorineural hearing loss (ISSHL) and presently applied treatments are off-label therapies. Current national clinical guidelines and an international consensus paper state that clinicians may offer systemic or intratympanic application of glucocorticoids for the initial treatment of ISSHL and intratympanic application is recommend when patients have incomplete recovery from sudden sensorineural hearing loss (DGHNO-KHC AWMF, 2014; Marx et al., 2018; Chandrasekhar et al., 2019). Intratympanic glucocorticoids may be applied as injections of solutions, with additives like hyaluronic acid gel, drug placed on pieces of absorbable gelatin sponge, applied *via* a Silverstein MicroWick™ or *via* an implanted catheter (Salt and Plontke, 2018). From several local application strategies, the most commonly used are single or repeated injections through the tympanic membrane. Based on available uncontrolled and controlled randomized and non-randomized studies, however, no clear recommendation can be made for specific application protocols (Liebau et al., 2017, 2018) and the evidence for the efficacy of intratympanic glucocorticoids for secondary (salvage) therapy of ISSHL through intratympanic injections is low (Plontke et al., 2022). Several aspects need to be considered for intratympanic therapy of inner ear disorders. (1) The round window membrane (RWM), shows various degrees of obstruction in one-fifth to one-third of the cases (Alzamil and Linthicum, 2000) with “blind” intratympanic injections involving the risk of the drug not reaching the RWM. (2) The time, the drug is in contact with the RWM is essential for the drug concentration in the inner ear perilymph (Hahn et al., 2006). Sustained drug delivery to the RWM can be realized through implantation of a catheter into the middle ear connected to an external pump (Kopke et al., 2001; Schwab et al., 2004; Plontke et al., 2009) or by polymers with controlled drug release properties, of which, a variety is currently being preclinically investigated for inner ear drug applications (Mäder et al., 2018; Salt and Plontke, 2018; Rathnam et al., 2019; Lehner et al., 2021). (3) The drugs that are mostly used for intratympanic therapies in humans - water-soluble forms like dexamethasone-phosphate – have pharmacokinetic properties that make them unsuitable for the therapy of some inner ear disorders. They are lost rapidly from the middle ear, enter perilymph slowly through the RWM, and in the inner ear, they are quickly dephosphorylated to the active drug form, but due to higher lipophilicity are then rapidly eliminated to the bloodstream (Salt and Plontke, 2018). (4) After a surgical tympanoscopy for exploration and removal of possible obstructions of the round window (RW) niche involving the risk of some blood in the middle ear and with a tympanomeatal flap requiring an ear canal dressing, repeated transtympanic injections are often not feasible for some time, suggesting the application of a drug depot or controlled release system in the RW niche at the time of surgery (Plontke, 2018). We previously reported on five patients with ISSHL, who were treated with an approved intravitreal implant for continuous dexamethasone application to the inner ear by implantation into the RW niche (Plontke et al., 2014). The rational for using such a drug delivery system was based on increasing knowledge on inner ear pharmacokinetics after local drug application and was thereafter individually offered to patients only for higher degrees of hearing loss (“non-serviceable hearing” or complete hearing loss on the affected ear) and planned tympanoscopy for inspection of the round and oval window niches.

We here report our experiences with this biodegradable drug delivery implant for controlled release of dexamethasone to the inner ear in a special therapeutic situation, i.e., tertiary therapy after failure of primary systemic application and secondary intratympanic injections of glucocorticoids.

## Materials and methods

### Study design, setting, and participants

The study is based on a retrospective chart review of patients treated for ISSHL at a single tertiary (university) referral center.

#### Inclusion criteria

Patients were included in this analysis if they received a tertiary (salvage) treatment of ISSHL with a solid, biodegradable, poly(D,L-lactic-co-glycolic acid) (PLGA)-based controlled release drug delivery system. Patients were offered this therapy as an individual treatment attempt for moderately severe, severe, or profound hearing loss (“non-serviceable hearing”) after unsuccessful primary systemic and secondary intratympanic (“blind” intratympanic injections) glucocorticoid therapy of ISSHL. The treatment was offered along with a tympanoscopy for inspection of the round and oval window niches for removal of possible RW obstructions and simultaneous tertiary local drug therapy through a “drug depot.”

#### Exclusion criteria

Patients with a known cause of the sudden sensorineural hearing loss [non-idiopathic hearing loss, e.g., Menière’s disease, suspected barotrauma, or vestibular schwannoma on magnetic resonance imaging (MRI)] were not offered such a treatment.

**FIGURE 1:**
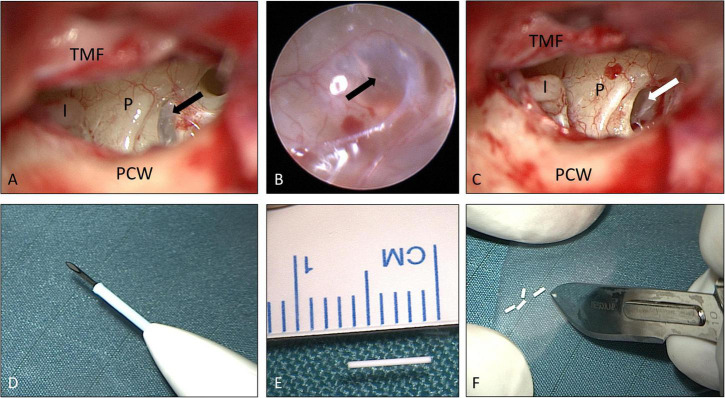
Surgical procedure: **(A–C)** tympanoscopy and removal of “false RWM” (patient No. 9, right ear). The black arrows show a mucosal membrane (“false RWM”) covering the RW niche. The white arrow shows the RWM after removal of a mucosal membrane. **(D–F)** Preparation of implant. The drug delivery system (0.46 × 6 mm) was unloaded **(E)** from the injection device for intravitreal delivery **(D)** and cut into approximately equal pieces of 1.5 mm each **(F)**. See also Supplementary video material. I: long process of incus (distal end); P: promontory; PCW: posterior canal wall; TMF: tympanomeatal flap. **(A,C–F)** Microscopic images; **(B)** endoscopic image, 1.7 mm, 30°). Figures **(A–C)** after Plontke (2018) with permission.

The various therapeutic options for salvage therapy of ISSHL through local delivery to the inner ear (see introduction) were discussed with the patient, and informed consent was obtained for this individual treatment regimen from each patient. The biodegradable drug delivery system was implanted in the RW niche. Due to increasing evidence of limited drug entry through the RWM, the last two patients in this case series received an intracochlear application of the drug delivery system through the RWM into the base of scala tympani. The implant (Ozurdex^®^, Allergan Inc., Irvine, CA, United States) is approved for intravitreal use and contains 0.7 mg dexamethasone in a PLGA polymer matrix containing a mixture of polymer chains with free and esterified carboxylic end groups without a preservative. It consists of the PLGA polymers RG502 und RG502H (RESOMER^®^, Evonik, Essen, Germany). RESOMER Evonik Healthcare (2022) the PLGA matrix slowly degrades to lactic acid and glycolic acid. The implant is 0.46 mm in diameter and 6 mm in length. For intravitreal application, the rod-shaped implant is injected from a pen-like device consisting of a hollow stainless-steel needle in a disposable applicator. For the adopted local controlled release drug delivery to the inner ear, the implant was removed from its applicator and cut into pieces of approximately 0.8–1.5 mm length, according to the size of the human RW niche (1.55 mm [range: 0.8–1.6 mm] or 1.65 mm [range: 0.96–2.28 mm] by 1.2 mm [range: 0.8–1.6 mm] (Su et al., 1982; Lang and Kothe, 1987; Figures 1D–F) and for implantation into scala tympani according to a recent study investigating feasible implant dimensions for intracochlear drug delivery in the human (Lehner et al., 2022).

### Surgery

**FIGURE 2:**
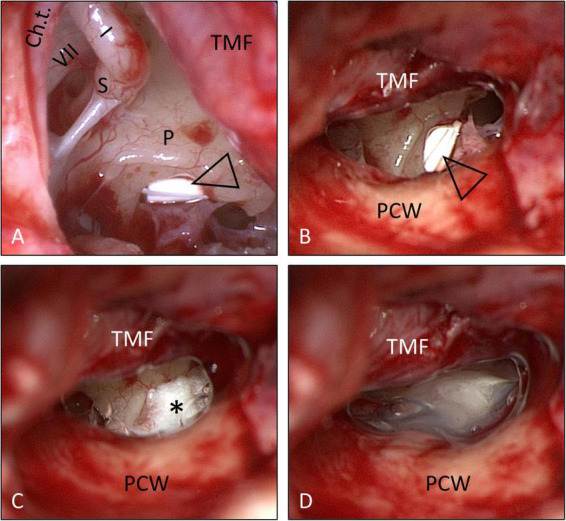
Surgical procedure (same patient as in Figure 1): Placement of the solid, biodegradable drug delivery system onto the RWM. **(A,B)** Implant pieces (open triangle) *in situ*. The implants were then covered with soft tissue (*) **(C)**, which was secured in places with fibrin glue **(D)**. See also Supplementary video material. Ch.t.: Chorda tympani; I: long process of incus; P: promontory; PCW: posterior canal wall; S: stapes head; TMF: tympanomeatal flap; VII: facial nerve. **(A)** Endoscopic view: 30°, 1.7 mm. Figure **(A)** after Plontke (2018) with permission.

**FIGURE 3:**
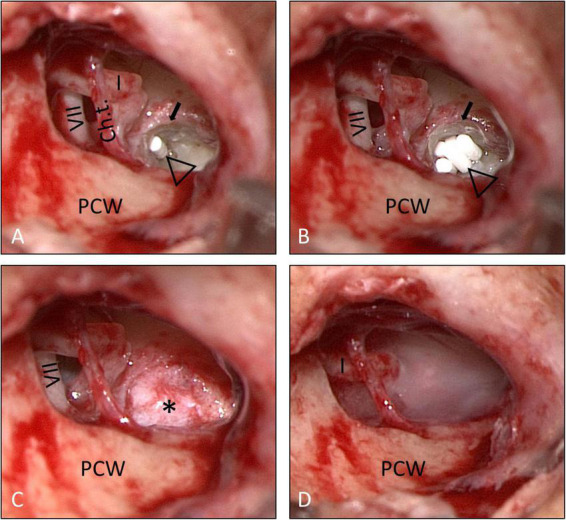
Surgical procedure (patient No. 14, right ear). **(A,B)** The bony rim of the RW niche was drilled away (small arrow) to allow a better view onto the entire RWM. The implants (open triangle) were then covered with soft tissue (*) **(C)**, which was secured in place with fibrin glue **(D)**. See also Supplementary video material. Ch.t.: Chorda tympani; I: long process of incus; PCW: posterior canal wall; VII: facial nerve.

**FIGURE 4:**
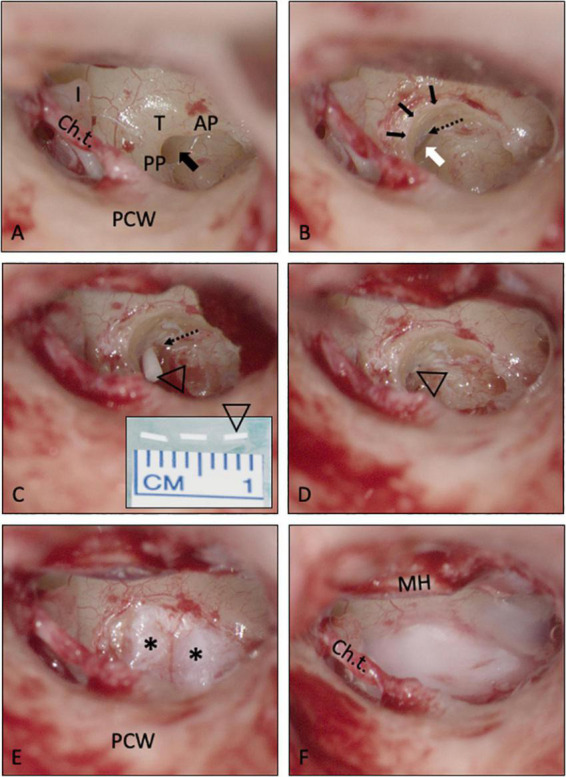
Intracochlear application of a solid biodegradable drug delivery system through the RW into scala tympani. **(A,B)** The RWM is usually hidden in the depth of the RW niche [black arrow in **A**; mean depth: 1.34 mm, range: 0.69–2.28 mm, (Su et al., 1982)]. To insert the drug delivery devise into scala tympani, the RWM (white arrow) was exposed by removing the bony rim of the RW niche [small black arrows in panel **(B)**], i.e., the tegmen (T), the anterior postis (AP) and partially also the posterior postis (PP). The RWM was opened, and the drug delivery system [open triangle in panels **(C,D)**] was gently pushed into the scala tympani with an otologic needle or hook. It was not necessary, to remove the crest of the RW, a thin vertical bony plate about 0.5 mm in height at the anterior border of the RW extending into scala tympani [dashed arrow in panels **(B,C)**]. The RW defect was sealed by covering the RW niche with soft tissue [* in panel **(E)**] and fibrin glue **(F)**. See also Supplementary video material. Ch.t.: Chorda tympani; I: long process of incus; MH: malleus handle; PCW: posterior canal wall.

Informed consent for surgery was obtained from all participants including off-label use of the controlled release implant (i.e., non-approved for this indication but for intravitreal use only) as an individual treatment attempt. The surgical procedure consisted of four steps: (1) tympanoscopy and removal of possible obstructions, (2) preparation and placement of the implant, (3) sealing with soft tissue and fibrin glue, and (4) ear canal dressing and wound closure. After raising a tympanomeatal flap, the middle ear and especially the oval and RW niches were inspected with the surgical microscope and an endoscope (1.7 mm, 30°). A possible partial or complete covering of the round niche by mucosal folds or membranes (often referred to as “false RWM”) was gently removed with a 0.4 mm 90° otosurgical hook (Figures 1A–C). After no signs of an oval or RW fistula were found, the controlled release implant pieces were transported into the RW niche with a 0.7 mm suction tip and placed onto the RWM (Figures 2A,B, 3A,B). Since RW niches are configured differently and the RWM is often only partially visible (Su et al., 1982; Tóth et al., 2006), it was necessary in some cases to drill away the bony rim of the RW niche (i.e., the tegmen, the anterior postis and partially also the posterior postis) to adequately inspect the entire RWM (black arrow in Figures 3A,B, 4B). In the first patients of this cases series, the implants were held in place with drops of fibrin glue. In later patients, we covered the implants with soft tissue, aiming at decreased loss of drug to the middle ear (Figures 2C, 3C). This was followed by application of fibrin glue (Figures 2D, 3D).

In the two patients with intracochlear application, the bony rim of the RW niche was drilled away to get access to the RWM. A slit-like opening was made in the RWM using an otologic 90°hook. This instrument was also used to gently push the rod-shaped implant into the base of scala tympani. The RW was sealed with soft tissue and fibrin glue (Figures 4A–F). For an illustration of the techniques see also Supplementary video material.

The tympanomeatal flap was then replaced, and a standard ear canal dressing was inserted. Dressing and sutures were removed 8–10 days after the procedure. The surgery can be performed under local anesthesia. In the presented cases, however, the patients requested general anesthesia. Some of the patients later received a cochlear implant (CI). In these cases, the RW niche and the basal part of scala tympani were inspected and material from the RW niche was recovered for histological examination before inserting the CI electrode array.

### Audiologic measurements

Audiological function was assessed preoperatively, 8–12 weeks postoperatively (follow-up 1), and 9–12 months after surgery (follow-up 2). Pure-tone and speech audiometry (German Freiburger monosyllables) were performed with an AT900 clinical audiometer (Auritec, Hamburg, Germany) and DT48A calibrated headphones (Bayer Dynamic, Berlin, Germany) in a sound-attenuated booth (Industrial Acoustics Company, Niederkrüchten, Germany). Ipsilateral pure-tone thresholds for air conduction were measured and reported as average across 0.5, 1, 2, and 4 kHz (4PTA). Word recognition score at 65 dB SPL (WRS_65_) and maximum speech discrimination (WRS_max_) were measured using lists of the Freiburger monosyllables test. The sound pressure level at WRS_max_ was reported as well.

According to the “research needs” stated in the “Clinical Practice Guideline: Sudden Hearing Loss” we also determined the percentage of patients who gained serviceable hearing after treatment. As emphasized there, we used “WRS percentages, acknowledging that even a severe pure-tone loss but with good or better word recognition ability is a good outcome” (Chandrasekhar et al., 2019).

### Neurotologic measurements

Due to the more invasive therapy, the two patients with intracochlear Ozurdex^®^ implantation received a detailed neurotological assessment including vestibular-ocular reflex (vHIT, ICS Impulse, Natus Medical Inc., San Carlos, CA, United States), cervical and ocular vestibular evoked myogenic potentials (c/oVEMPs, Eclipse, Interacoustics, Middelfart, Danmark) and response to caloric stimulation (Hortmann Vestlab 100, Natus Medical Inc., San Carlos, CA, United States).

### Histology

Material for histological examination was gained from the RW area in patients later receiving a cochlear implant. Tissue sections from formalin-fixed, paraffin-embedded tissue were stained with standard hematoxylin-eosin (H&E, Carl Roth, Karlsruhe, Germany). In addition, an iron stain (“Prussian blue,” pretreatment with hydrochloric acid 5%, Carl Roth, Karlsruhe, Germany) followed by potassium-hexacyanoferrate (II) trihydrate, 1:100, Sigma-Aldrich) was performed to visualize blood residua. To perform immunohistochemistry, the slides were pretreated with enzyme (BOND Enzyme Pretreatment Kit, Leica Biosystems, Nussloch, Germany) for 10 min for antigen retrieval. CD68 immunohistochemistry (clone KP1 1:100, Dako, Agilent Technologies, Santa Clara, CA, United States) was utilized to label macrophages of patients No. 11 and 13. Cytokeratin Pan Plus (PanCKplus) immunohistochemistry (pH 9 for 10 min, BMS057, Zytomed Systems, Berlin, Germany) was used to label the epithelial surface.

### Statistics

Averaged pure-tone thresholds (4PTA) and speech perception scores (WRS_65_, WRS_max_) were descriptively analyzed and compared between the initial and follow-up visits by paired one-tailed *t*-tests using SPSS software (IBM, Armonk, NW, United States). Alpha was set to 5%. Influence of treatment delay on hearing gain and final 4PTA at follow-up was descriptively analyzed by spearman regression using SPSS. Alpha was set to 5%.

### Mathematical simulations of drug distribution

All simulations of glucocorticoid distribution in the perilymph of scala tympani were performed with FluidSim V4.05^1^ over a period of 14 and 35 days, respectively. For intratympanic injection, 0.3 ml of a 4 mg/ml dexamethasone sodium phosphate solution was chosen (injected once every 2 days, with a pause of 3 days [e.g., on the weekend] and five injections in total), while 20 min after each application we assumed an almost total drug loss from the middle ear due to the clearance over the Eustachian tube. For extracochlear application of Ozurdex^®^ (total dexamethasone: 700 μg), the applied volume to the middle ear was set to 10 μl with zero volume clearance because of covering of Ozurdex^®^ with soft tissue. For simulations of intracochlear delivery, 1/2 Ozurdex^®^ implant (i.e., 350 μg dexamethasone) was assumed to be placed 1 mm behind the RWM in the basal region of scala tympani. The simulations were based on Ozurdex^®^ *in vitro* drug release kinetics (Bhagat et al., 2014; Lehner et al., 2019). All other parameters remained at the program’s default values.

### Ethical approval

Data collection from patient charts, data analysis, and reporting of outcome after therapies for sudden hearing loss were approved by the ethics committee of the Martin-Luther University of Halle-Wittenberg (approval number 2019-109, extended in 2022).

## Results

### Participants

Between 2011 and 2021, 17 patients were implanted with the biodegradable drug delivery system for controlled dexamethasone release in a tertiary salvage therapy setting (extracochlear into the RW niche: *n* = 15; intracochlear into scala tympani: *n* = 2). All patients were initially treated according to the German AWMF (working group of medical scientific societies) guideline with high-dose intravenous prednisolone (250 mg/d over 3 days with subsequent dose reduction) starting within 2 days after ISSHL followed by “blind” intratympanic injections of dexamethasone phosphate (Fortecortin 4 mg/mL, Merck Darmstadt, Germany; daily over 5 days) as secondary (salvage) therapy. Demographic and patient characteristics are described in detail in Table 1. One patient (patient No. 2) received hyperbaric oxygen therapy parallel to intratympanic injections before tertiary therapy.

**TABLE 1:** Summary of demographic data, medical conditions, and intraoperative findings at tympanoscopy.

No.	Sex	Age	Side	Days after ISSHL		RWM obstruction	Secondary diagnoses
						
				Start secondary therapy	Start tertiary therapy (implantation)		
1	M	64	R	9	13	Partial	CHD with St. p., CABG, hypertension, IDDM, hyperlipoproteinemia
2	F	47	R	11	16	na	None known
3	M	74	R	7	18	None	St.p. vertebrobasilar brain infarct some years ago; aHTN, fat metabolism disorder, CHD, Glaucoma
4	M	68	R	21	29	None	Newly diagnosed IDDM; sever congenital amblyopia
5	M	79	R	26	27	Total	CHD with St.p. MI some years ago, aHTN, hyperuricemia, mild pulmonary and aortic valve disease
6	M	54	R	15	23	Subtotal	CHF with St.p. MI some years ago, aHTN, OSAS, metabolic syndrome, hypertriglyceridemia
7	M	75	R	11	33	Partial	aHTN
8	M	61	L	19	26	Total	aHTN, hypothyreosis after thyroidectomy, migraine headache
9	M	61	R	90	130	Total	None known
10	M	51	L	12	17	None	None known
11	F	47	L	30	42	Total	None known
12	M	64	L	8	20	Total	None known
13	F	47	R	>15	>30	Subtotal	Cochlear Hydrops in MRI without vertigo
14	F	50	R	27	45	Total	Recurrent sudden hearing loss; hypothyreosis
15	F	51	L	8	11	None	None known
16	M	49	L	6	33	Total	None known
17	F	75	R	8	36	None	Rheumatoid arthritis
Mean (±SD)	6F/11M	60 (±20)	11R/6L	19 (±19)	32 (±26)		

No. indicates patient number; M, male; F, female; aHTN: arterial hypertension; CABG: coronary artery bypass graft; CHD: coronary heart disease; ICA: internal carotid artery; IDDM: insulin dependent diabetes mellitus; MI: myocardial infarction. na: not available; OSAS: obstructive sleep apnea; St. p.: status post. Patient No. 1 from Plontke et al. (2014).

### Surgical aspects

Implantation of the Ozurdex^®^ rods was feasible in all patients. While extracochlear placement of the implants onto the RWM was possible with a small rim of the RW niche remaining, it was necessary to drill away the tegmen, the anterior postis and partially the posterior postis of the RW niche to the level of the RWM in the two cases, where the implant was inserted in to scala tympani (Figures 4A,B). To insert the implant, however, it was not necessary to remove the crest of the RW (the thin vertical bony plate about 0.5 mm in height at the anterior border of the round window extending into scala tympani [Tóth, 2019)].

On tympanoscopy, partial or complete (total) obstructions of the RW niche were found in the majority of patients (11/16; 69%, data not available in one patient). In detail: a partial obstruction of the RW niche was found in two; a subtotal obstruction in two, a total obstruction by mucosal tissue was found in seven patients and five patients showed no obstruction of the RW niche.

**FIGURE 5:**
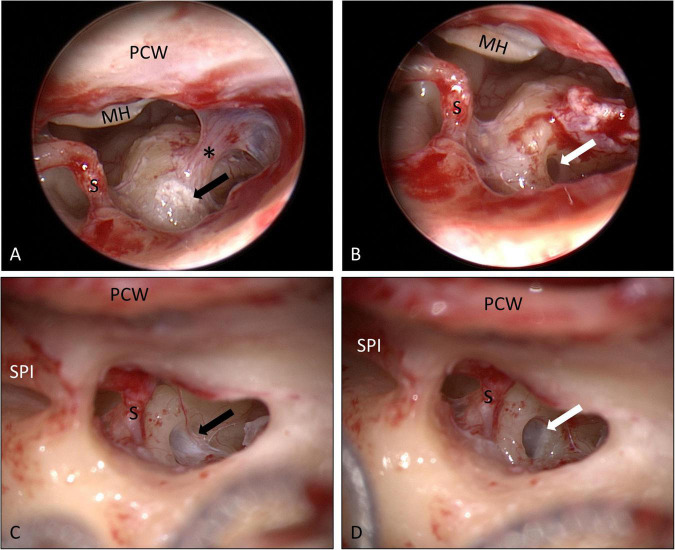
Round window niche area as seen through a posterior tympanotomy at the time of cochlear implantation 18 months **(A,B)** and 16 months **(C,D)** after implantation of the biodegradable drug delivery system and sealing with soft tissue. Mild scarring (*) between the promontory and the tympanic membrane was found (**A**) and cut. Some fibrous tissue was found in the RW niches [black arrows in panels **(A,C)**] that could be removed without damaging the RWM [white arrows in panels **(B,D)**]. The white material in panel **(A)** corresponds to calcifications. No residuals of the drug delivery implants were found (see also histology of these patients in Figures 6B,D, and immunohistochemistry in Figures 7A–C). MH: malleus handle; PCW: posterior canal wall; S: stapes head; SPI: short process of incus. **(A,B)** endoscopic images, 0°, 4 mm; **(C,D)** microscopic images.

In patients, who later received a cochlear implant, small amounts of fibrous tissue were found in the RW niche (Figure 5). In one patient, we found scarring between the promontory and the tympanic membrane (Figure 5A). However, the fibrous tissue could be removed without complications revealing a completely inconspicuous RWM (Figures 5B,D). There were no other abnormalities seen in the RW area, at the promontory or the other bony structures of the middle ear. In five patients (29%), the tympanic membrane appeared thin in the lower inferior quadrant, likely due to the prior intratympanic injections for the secondary therapy of ISSHL. The tympanic membrane was therefore reinforced in underlay technique with some soft tissue from the endaural incision site. Apart from expected mild postoperative pain at the incision side, no adverse events like persistent pain, middle ear inflammation, vertigo, eardrum perforation or facial paralysis were observed.

### Histologic assessment and magnetic resonance imaging

**FIGURE 6:**
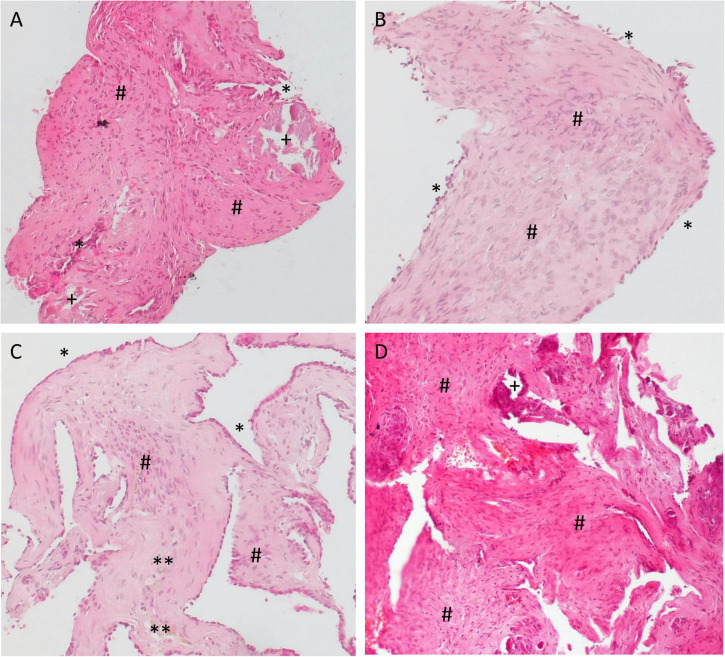
Histology examination of specimens taken from the RW area of different patients at the time of cochlear implantation. **(A,B)** 16 months; **(C)** 6 months, and **(D)** 18 months after implantation of the biodegradable drug delivery system and sealing with soft tissue. Hematoxylin-eosin (H&E), 100x. Histomorphology was comparable between patients. Particles of collagenous fibers with a moderate cellularity were covered on the surface with one layer of a monomorphic flat to cuboid epithelium (*). The stroma contained areas of resting, as well as of proliferating fibroblasts (#) and some macrophages (**). There was no substantial inflammation. In some specimen, we found scattered, medium sized amorphous calcifications (+) within the stroma that did not react to polarized light.

**FIGURE 7:**
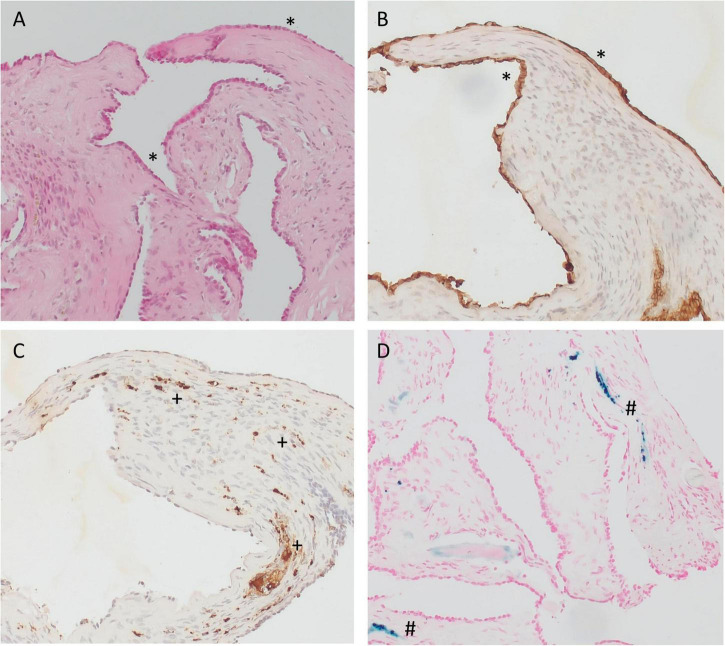
Immunohistochemistry of specimens taken from the RW area at the time cochlear implantation. **(A–C)** 16 months and **(D)** 6 months after implantation of the biodegradable drug delivery system and sealing with soft tissue. **(A)** hematoxylin-eosin (H&E), 100x; **(B)** PanCKplus, 100x; **(C)** CD68, 100x; **(D)** Prussian blue, 100x. (Immuno-)histochemistry confirmed that the surface cover (*) was a true epithelium (PanCKplus positive), **(A,B)**. Within the stroma, we found scattered CD68-positive macrophages (+) as a sign of chronic resorptive inflammation **(C)** and/or tissue reactivity to Prussian blue (#) as a sign of blood residua **(D)**. **(A**–**C)** Same patient as in Figure 6B; **(D)** same patient as in Figure 6C.

Specimens from the RW area were taken 6–18 months after implantation of the biodegradable drug delivery system during cochlear implantation in five patients (*n* = 4 after extracochlear, *n* = 1 after intracochlear application of the drug delivery system). Histomorphology showed comparable features in all patients. We saw particles of collagenous fibers with a moderate cellularity, covered on the surface with one layer of a monomorphic flat to cuboid epithelium. The stroma contained predominantly areas of resting and proliferating fibroblasts. There was no substantial inflammation or foreign body reaction. Depending on the duration of time after surgery, the stroma contained a variable amount of scattered CD68-positive macrophages as a sign of chronic resorptive inflammation. As time after surgery increased, the number of macrophages decreased. Furthermore, the tissue showed reactivity to Prussian blue as a sign of blood residua. In a part of the specimen, we saw scattered, medium sized amorphous calcifications within the stroma that did not react to polarized light (Figures 6, 7).

**FIGURE 8:**
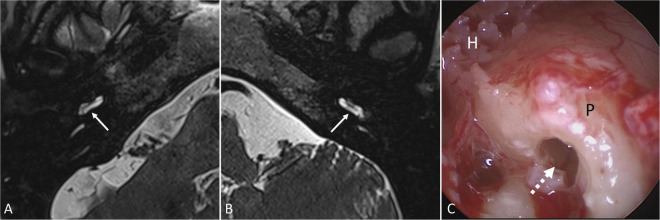
Magnetic resonance imaging (T2-weighted 3D-SPACE, axial, 0.4 mm) showing the basal cochlear turn of patient No. 17 **(A)** 6 months and of patient No. 16 **(B)** 9 months after intracochlear implantation of Ozurdex^®^ into the basal part of scala tympani. Regular fluid signals were found in the scala tympani of the basal turn (→) and in the entire inner ear without pathologic contrast enhancement and without signs of implant residuals (Department of Radiology, University Medicine Halle, Sabrina Kösling with permission). On cochlear implantation of patient No. 16, no signs of inflammation, foreign body reaction or residuals of the implant were found in the basal part of scala tympani (dashed arrow) **(C)**. H: hypotympanon; P: promontory.

Magnetic resonance imaging was performed in six patients (*n* = 4 after extracochlear, *n* = 2 after intracochlear application of the drug delivery system) as preoperative diagnostic before cochlear implantation to ensure scalar patency for CI array insertion. In these patients, regular fluid signals were found in the entire inner ear without pathologic contrast enhancement. No signs of residuals of the implant were detected in the cochleae for the two patients with intracochlear application (Figure 8). In all patients with later cochlear implantation, the CI array could be inserted without problems, i.e., without any resistance.

### Neurotologic assessment

In patient No. 16 with intracochlear application of the drug delivery system and with preoperative vertigo, neurotologic assessment preoperatively showed a regular caloric response but reduced gain in the vHIT test of the lateral and posterior canals with overt saccades and absent oVEMPs and cVEMPs in the ear affected by ISSHL. Postoperatively, the gain improved to normal values in the lateral and remained reduced in the posterior canal with few scattered overt saccades, but absent caloric response. Symmetrical, low amplitude cVEMP and oVEMP responses were recorded postoperatively. In patient No. 17 with intracochlear application of the drug delivery system and without preoperative vertigo, postoperative measurements showed normal vHIT gain without covert or overt saccades in all semicircular canals, a regular caloric response, and symmetrical, low amplitude cVEMP and oVEMP responses.

### Audiologic assessment

**FIGURE 9:**
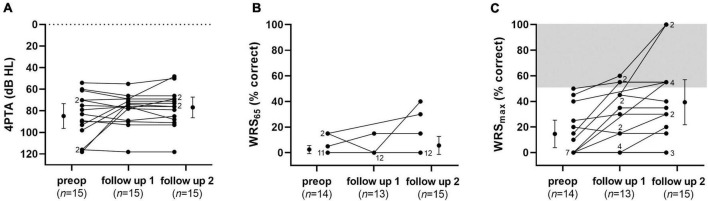
Audiological results: Pure-tone thresholds **(A)**, word recognition scores at 65 dB SPL **(B)** and maximum word recognition scores **(C)** preoperatively, 8–12 weeks, and 9–12 months after implantation of the drug delivery system with controlled release of dexamethasone into the RW niche as individual values and means (preoperative and follow-up 2). Error bars show the 95% confidence interval. Numbers indicate the amount of superposed data points.

**FIGURE 10:**
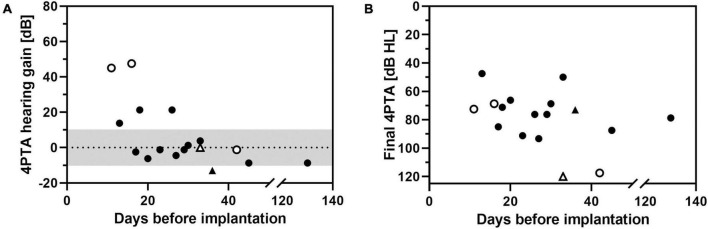
Change in pure-tone average (4PTA hearing gain, **A**) and final hearing threshold at follow-up 2 (final 4PTA, **B**) as a function of time of implantation (days after onset of ISSHL). Triangles: intracochlear application of the drug delivery system. Open symbols: non-measurable threshold (out-of-limits) at time of implantation.

Audiological assessments are shown in Figures 9, 10 and Table 2. The average pure-tone threshold was 84.7 dB HL (SD: 20.0) preoperatively, 78.2 dB HL (SD: 14.1) 8–12 weeks, and 76.7 dB HL (SD: 16.7) 9–12 months post implantation. The average word recognition score at 65 dB SPL (WRS_65_) was 2.5% (SD: 5.3) preoperatively, 1.2% (SD: 4.0) 8–12 weeks, and 5.7% (SD: 12.2) 9–12 months post implantation. Average maximum speech discrimination (WRS_max_) was 14.6% (SD: 17.9) preoperatively, 27.3% (SD: 22.5) 8–12 weeks, and 39.9% (SD: 30.7) 9–12 months after implantation. None of the differences was statistically significant. At 9–12 months after start of tertiary therapy of ISSHL with the biodegradable implant, 6 of 15 (40%) of the patients with extracochlear (RWM) application reached “serviceable hearing” (>50% word recognition score), i.e., the ear would be a candidate for traditional hearing amplification according to the criteria of the American clinical practice guideline (CPG) (Chandrasekhar et al., 2019). This improvement may thus be rated as partial, but clinically relevant improvement (for maximum WRS versus WRS at 40 dB Sensation level [SL] see the section “Discussion”). According to the German cochlear implant guideline, only two patients (13%) reached a word recognition score better than the indication criteria for a CI (>60% monosyllables at best aided condition or WRS_max_, respectively) (DGHNO-KHC AWMF, 2020). One of these patients (No. 13) deteriorated later and received a cochlear implant. None of the two patients with intracochlear implantation of the drug delivery system reached serviceable hearing. One of these two patients (No. 17) deteriorated slightly with respect to 4PTA (−13 dB) and WRS_max_ (−10%), while the WRS at normal speech level of 65 dB SPL improved from 15 to 35%. None of the patients of the entire group (*n* = 17) showed a WRS_65_ of ≥65% at normal speech level (65 dB SPL), i.e., useful hearing without a hearing aid at 9–12 months post implantation.

**TABLE 2:** Summary of audiologic outcome data.

No.	4PTA (dB HL)			WRS_65_ (%)			WRS_max_ (%) (at dB SPL)			Recovery
				
	pre	f/u1	f/u2	pre	f/u1	f/u2	pre	f/u1	f/u2	
**Extracochlear application (RW niche)**										
1	61	69	48	na	0	40	na	45 (100)	100 (95)	partial
2	116	78	69	0	na	0	0 (110)	na	55 (100)	partial
3	93	71	71^§^	5	15	15^§^	15 (80)	55 (95)	55 (95)^§^	partial
4	75	76	76^§^	0	0	0^§^	20 (95)	15 (95)	15 (95)^§^	no
5	89	93	93^§^	0	0	0^§^	0 (80)	0 (100)	0 (100)^§^	no
6	90	90	91	0	0	0	0 (80)	0 (100)	0 (110)	no
7	54	55	50	15	na	30	40 (80)	na	55 (80)	partial
8	98	74	76	0	0	0	0 (80)	15 (95)	30 (95)	partial
9	70	78	79	0	0	0	0 (80)	0 (80)	20 (100)	partial
10	83	89	85	0	0	0	25 (95)	30 (110)	30 (100)	no
11	116	118	118	0	0	0	0 (100)	0 (95)	0 (95)	no
12	60	66	66^§^	15	0	0^§^	50 (95)	55 (95)	55 (95)^§^	no
13	70	69	69	0	0	0	45 (95)	60 (95)	100 (95)	partial*
14	79	76	88	0	0	0	10 (80)	45 (95)	40 (95)	partial
15	118	73	73	0	0	0	0 (100)	35 (95)	35 (95)	partial
*Mean*	84.7	78.2	76.7	2.5	1.2	5.7	14.6	27.3	39.3	
*SD*	20.0	14.1	16.7	5.3	4.0	12.2	17.9	22.5	30.7	
**Intracochlear application (scala tympani)**										
16	120	120	120	0	0	0	0 (110)	0 (110)	0 (110)	no
17	60	74	73*	15	0	35*	45 (110)	15 (110)	35 (110)	no

f/u1 and f/u2: follow-up 8–12 weeks and 9–12 months after implantation, na: not available; §: last value (f/u1) carried forward. 4PTA: (pure-tone average from 0.5 to 4 kHz, WRS_65_: (%) of monosyllables correctly understood at 65 dB SPL in quiet, WRS_max_ (%) at (dB): maximum number of monosyllables understood in quiet (in%) at dB SPL value in parenthesis. *Patient No. 13 deteriorated again later and then received a cochlear implant. Patient No. 1 from Plontke et al. (2014). *Patient o. 17: f/u2 measurement 6 months after ISSHL.

A correlation between higher hearing gain with earlier start of treatment after onset of ISSHL was found (*R*^2^ = 0.615, *p* = 0.015, Figure 10A). When final 4PTA at follow-up was chosen as outcome parameter, this correlation was not present (Figure 10B). No hearing improvement of >10 dB was noticed in patients who received a tertiary therapy after 30 days of onset of ISSHL.

### Mathematical simulations

**FIGURE 11:**
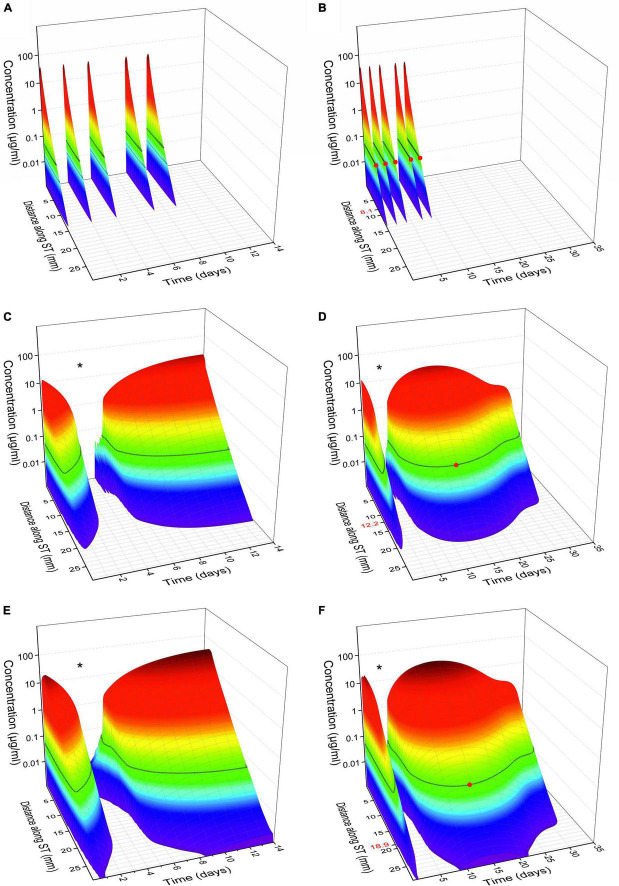
Calculated glucocorticoid distribution in the perilymph of scala tympani (ST) over 14 and 35 days, respectively. **(A,B)** Intratympanic injection of 0.3 ml of a 4 mg/ml dexamethasone phosphate solution (once every 2 days, with an assumed pause of 3 days on the weekend, 5 injections in total). **(C,D)** Extracochlear application of the Ozurdex^®^ implant (total dexamethasone: 700 μg) on the RWM. **(E,F)** Intracochlear application of 1/2 Ozurdex^®^ implant (350 μg dexamethasone) in the basal region of scala tympani. The red dot on the black line in panels **(B,D,F)** and the corresponding red number on *y*-axis indicate the farthest apical spread when assuming a minimal therapeutic concentration of 50 ng/ml dexamethasone for inflammatory suppression (horizontal black lines on surface plots) (Liu et al., 2015, 2016; Bas et al., 2016; Liebau et al., 2020). The “dip” in the intracochlear concentration-time-distance curves [* in panels **(C–F)**] can be explained by the plateau in the *in vitro* dexamethasone release kinetics of the drug delivery system (see Figure 12). When comparing the concentration time curves, it needs to be considered that dexamethasone phosphate **(A,B)** and dexamethasone **(C–F)** differ in their different molecular properties (Salt and Plontke, 2018).

Mathematical simulations of intracochlear drug concentrations showed that – in comparison to intratympanic injections – the administration of the biodegradable Ozurdex^®^ implant in the RW niche leads to constant and sustained dexamethasone concentrations in the cochlear perilymph over several weeks (Figures 11A–D). Despite a long residence time of the drug in the RW niche, however, only very limited drug concentration is expected in the apical region (Figures 11C,D). Simulations of drug concentrations using the biodegradable Ozurdex^®^ as an intracochlear implant showed higher maximum concentrations and a more apical spread of dexamethasone (Figures 11E,F).

## Discussion

### Rationale and challenges of different types of intratympanic drug application

The rationale behind intratympanic drug delivery to the inner is that drug can diffuse through the RWM (and likely the oval window) into the inner ear and from there spread to the different parts of the inner ear tissues. Advantages include (1) bypassing the blood perilymph barrier (Glueckert et al., 2018; Salt and Hirose, 2018; Nyberg et al., 2019), (2) higher drug levels in the inner ear than with systemic application (e.g., for dexamethasone demonstrated in both, animals, and humans: Bird et al., 2011; Lee et al., 2018), (3) lower total amount of drug given, (4) avoiding “first pass effects,” (5) reduced risks from possible systemic side effects, and (6) specific targeting of the inner ear organ.

Intratympanic injections are widely used in clinical practice. Although this procedure is of rather low invasiveness, it has some drawbacks. One disadvantage is the rapid clearance of the injected drug solution from the middle ear *via* the Eustachian tube. Secondly, only small amounts of drug reach the inner ear by diffusing through the RWM, which exhibits a relevant diffusion barrier. Additional obstructions of the RW niche can impede the entry of drugs into the cochlea (Alzamil and Linthicum, 2000). Substances that have passed the RWM are distributed in the perilymph by diffusing toward the apical regions of the cochlea but are simultaneously absorbed by the surrounding tissue of the scala tympani. Depending on the physicochemical properties of the drug used (diffusion characteristic, lipophilicity), a basal to apical concentration gradient results in the cochlear fluids (Figures 11A,B; Salt and Plontke, 2018). To maximize the concentration reaching the apical regions of the inner ear, the initial drug concentration at the base of the cochlear should be as high as possible.

By extending the time the drug is in contact with the RWM, higher drug concentrations can be achieved over a longer period, which is especially beneficial for drugs with fast clearance in the perilymph, such as glucocorticoids [elimination half-time of 22.5 min for dexamethasone (Salt et al., 2012)]. To increase the residence time on the RWM, hydrogels have been suggested as drug carriers, in particular, the thermosensitive Poloxamer 407 (Piu et al., 2011) and hyaluronic acid (El Kechai et al., 2016). Another strategy to prolong drug release to the inner ear is the invasive implantation of drug depots directly in front of the RWM in the RW niche. Microcatheters such as the Silverstein MicroWick™ or the Round Window μ-Cath™ are intended to ensure a higher and more even distribution of drugs in the inner ear, but must be surgically removed after the end of therapy (Silverstein et al., 2004). Biodegradable drug depots show advantages in avoiding invasive (surgical) procedures for removal (Plontke et al., 2014; Lehner et al., 2021). Mathematical simulations suggested constant and sustained dexamethasone concentrations in the cochlear perilymph over several weeks after administration of the biodegradable implant in the RW niche (Figures 11C,D).

In addition, possible obstructions of the RW (e.g., “false RWMs”) can be removed at the time of implantation. In our case series, partial or complete obstructions of the RW niche were found in 11 of 16 patients, which is larger than thus reported by others (Alzamil and Linthicum, 2000). While the high rate of obstructions might be biased due to a small sample size it may also be explained by a “negative selection” of patients, with the obstructions having potentially contributed to an unsuccessful secondary therapy (“blind” intratympanic injections).

### Safety aspects and audiologic outcome

A tympanoscopy (middle ear exploration) is a standard otological procedure and has been applied for many years in combination with “sealing” of the RW niche in patients with ISSHL (Loader et al., 2013; Kampfner et al., 2014) and for removal of possible RWM obstructions in patients with ISSHL (Plontke, 2018) or with Meniere’s disease (Crane et al., 2009) who have failed therapy with (“blind”) intratympanic injections. All patients in our institution, who this individual treatment attempt was offered to, had quasi lost a sensory organ (complete hearing loss or non-serviceable hearing in the affected ear) and failed other guideline-conform therapies. A successful therapy (“serviceable hearing”) avoids cochlear implantation for hearing rehabilitation which would involve many more costs and more extensive surgery. Thus, we consider the benefit-risk ratio for the surgical intervention in general to be favorable.

The drug delivery system used in the present study is completely biodegradable by ester cleavage through hydrolysis upon contact with water (Park, 1994; Li and Vert, 2002). Lactic and glycolic acid are incorporated into the tricarboxylic acid cycle and subsequently excreted (Athanasiou et al., 1996). Clinical observation, audiological evaluation, histological examination, MRI and neurotologic assessment (where available) showed no signs of adverse events. No threshold deterioration was observed apart from one patient (No. 17 with Δ4PTA = −13 dB but improvement in speech audiometry with ΔWRS_65_ of +20%). In the small number of patients, where inspection of the RW niche was later possible during cochlear implantation, the Ozurdex^®^ implant was no longer visually detectable, confirming the theoretical degradation time of 3 months for this kind of polymer (RESOMER Evonik Healthcare, 2022). Histologic examination of the material in the RW niche showed signs of resorption and healing without relevant inflammation and without foreign body reaction. Thus, the procedure appeared to be safe.

Our case series showed mixed results with respect to audiological outcome. We observed a significant improvement in 4PTA and WRS_max_ in patients with an early intervention and 40% of the patients with extracochlear Ozurdex^®^ implantation into the RW niche reached serviceable hearing according to the American CPG, and 13% according to the German CI guideline, respectively (Chandrasekhar et al., 2019; DGHNO-KHC AWMF, 2020). However, some uncertainties in this respect must be considered, since German monosyllabic words from the Freiburger test list are somewhat more difficult than two-syllabic spondaic words and word recognition score at 40 dB SL, which is regularly used in the United States, is not equal to the maximum word recognitions WRS_max_ as typically used in Germany (Plontke et al., 2009; Gurgel et al., 2012; Müller et al., 2017; Hoppe et al., 2019).

Mean hearing improvement was in the range also observed in other studies with patients showing similar degrees of hearing loss before intratympanic salvage treatment of ISSHL (Liebau et al., 2018). However, in two of three patients with profound hearing loss of 4PTA > 100 dB HL, hearing improvements of more than 40 dB were observed. In a clinical pilot study by Plontke et al., with a secondary (salvage) treatment of ISSHL using another type of continuous drug application to the RW [a RW μ-Cath delivering dexamethasone phosphate over 14 days (Plontke et al., 2005)], seven patients with hearing loss > 100 dB HL were included with two patients showing a hearing improvement of more than 40 dB. In a randomized controlled clinical trial, dexamethasone phosphate was also applied by a RW μ-Cath over 14 days as a secondary (salvage) treatment (Plontke et al., 2009). Among the included patients in the intervention arm, five had hearing loss > 100 dB HL and in two of them, hearing improved more than 40 dB. The percentage of patients with profound hearing loss in the present study showing a hearing gain > 40 dB was therefore slightly higher than seen in these previous RW catheter studies. However, compared to the reported hearing recovery in the literature (Liebau et al., 2018) there is no apparent increase in treatment success with RW implantation of Ozurdex^®^.

Patients, who received an earlier start of treatment after onset of ISSHL showed a correlation toward a greater hearing gain (Figure 10A). This trend has also been reported using intratympanic glucocorticoid injections (Liebau et al., 2017). However, in the meta-analysis of Liebau et al. the tendency toward a positive effect of early treatment on hearing gain was interpreted as a “sham effect.”

### Rationale and challenges of intracochlear drug application

Several strategies are under investigation to enhance drug entry into the inner ear through the RWM using magnetic fields (Du et al., 2013; Shapiro et al., 2014), ultrasound (Lin et al., 2021), RWM microperforations (Aksit et al., 2021), or nanocarriers (Jaudoin et al., 2021). Approaches to overcome or at least decrease intracochlear basal-apical concentration gradients involve the application of RWM low-frequency micro vibrations (Flaherty et al., 2021) or direct intracochlear drug application (Chen et al., 2005; Hahn et al., 2012; Ayoob and Borenstein, 2015; Plontke et al., 2017; Prenzler et al., 2018). In a clinical pilot study, an intracochlear catheter was inserted into the cochlea during CI surgery before insertion of the electrode array to deliver triamcinolone acetonide (Prenzler et al., 2018, 2020). Patients who received high dose triamcinolone acetonide (20 mg/ml) showed lower impedances compared to a control group (with no steroid application) or to a low dose group (4 mg/ml), however, the effect only lasted 4 weeks after application.

The electrode arrays CI632D (Cochlear, Sydney, Australia) and CIDEXEL (MED-EL, Innsbruck, Austria) are loaded with dexamethasone to suppress insertion trauma after cochlea implantation, and are used in clinical trials (Briggs et al., 2020; ClinicalTrials, 2022). Briggs et al. described lower impedance values lasting over the whole observation period of 2 years for the dexamethasone-eluting device in contrast to drug-free electrode arrays (Briggs et al., 2020). The fixed combination of the electrode array with an incorporated drug, however, limits possibilities for personalized treatment. A drug delivery system separated from the electrode array is desirable because both systems can be optimized independently. This creates new opportunities to personalized medicines as suggested previously (Plontke et al., 2017; Lehner et al., 2022). Although the intracochlear application of drugs is likely associated with higher risks than intratympanic application (Salt and Plontke, 2009) the above studies on intracochlear glucocorticoid drug application did not report relevant side effects.

**FIGURE 12:**
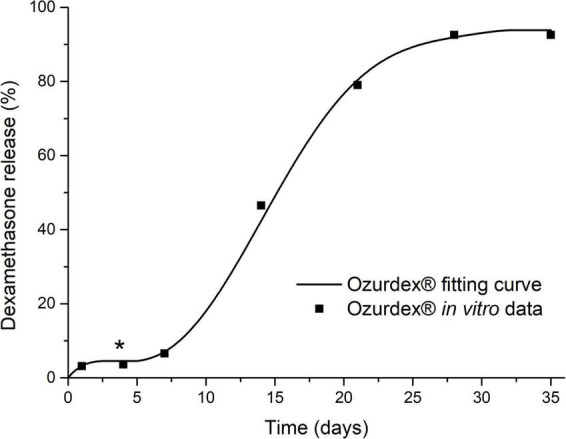
Dexamethasone release kinetics of the drug delivery system Ozurdex^®^ [*in vitro* relative cumulative release according to Bhagat et al. (2014)] which was used for the simulations in Figure 11. The plateau phase (*), i.e., zero release, explains the “dip” in the intracochlear concentration-time-distance curves in Figures 11C–F.

When using Ozurdex^®^ as an intracochlear implant, mathematical simulations showed higher maximum concentrations and a more apical spread of dexamethasone compared to RW application (Figures 11B–D). The material characteristics of the Ozurdex^®^ implant, do not meet the desired requirements for implantation into the inner ear in a perfect way, and its drug release profile shows suboptimal drug levels over several weeks (Figure 12; Bhagat et al., 2014; Lehner et al., 2019). Drug delivery systems for intracochlear drug application are the focus of preclinical research. Pierstorff et al. (2018) developed polyvinyl alcohol-coated fluticasone propionate particles that can be injected through the RWM into the scala tympani. In contrast to larger solid implants, the intracochlear application of these particles may be less traumatic. However, polyvinyl alcohol is not biodegradable, and it is unclear how the polymer is eliminated from the inner ear. Another idea to deliver glucocorticoids directly into the cochlea are sharpened dexamethasone-loaded PLGA implants, which can puncture the RWM and be positioned in the scala tympani (Pawley et al., 2021). *In vivo* data showed the general feasibility of its administration and geometrical properties, however, they approach seems improvable with regard to damage to the RWM. Like in the Ozurdex^®^ implant, release kinetics of these sharpened PLGA implants are suboptimal showing no homogenous release and an initial lag-phase without drug release. In addition, information is missing if residual solvents are remaining in the implants due to the manufacturing process. Lehner et al. developed a PLGA based drug depot with an improved, homogenous drug release profile over more than 5 weeks and smoother material characteristic which has already been tested in human temporal bones (Lehner et al., 2019, 2022). In PLGA-based implants, including Ozurdex^®^, the free acids emerging during degradation can change the pH microclimate inside and around the implant (Siepmann et al., 2005; Schädlich et al., 2014). Although endogenous bicarbonates buffer the perilymph, toxic effects on the hair cells cannot be ruled out. Further studies are therefore required to consider possible pH change of perilymph in the development of such drug depots. Since experience with biodegradable drug delivery systems for the controlled release of substances to the inner ear after extracochlear or intracochlear application in the human are very rare, our observations may be helpful for the use of such systems in clinical trials for therapy of inner ear disorders and for the development of future drug delivery systems.

### Limitations of the study

Although we did not observe any signs of adverse events or even toxicity in our patients, the number of patients where we were able to inspect the RW niche after a longer follow-up period, is small. Due to the lack of a control group, the study design is not suited to adequately answer the question of efficacy with respect to hearing outcome. Prospective, controlled studies are necessary to address this question. In our study, patients with a degree of sudden hearing loss, that is associated with a poor prognosis, were offered this treatment in a special therapy situation, i.e., after insufficient recovery after both, systemic primary therapy and secondary intratympanic injections on an individual basis due to their non-serviceable hearing. This special clinical situation also explains the variability in the delay between ISSHL and tertiary salvage therapy. It is unclear, however, whether there exists a critical therapy window after onset of sudden hearing loss. Since patients received the intervention as a tertiary salvage treatment, the critical therapy window may already have elapsed in some or most of the included patients. In addition, there is still uncertainty, which types of (idiopathic) sudden sensorineural hearing loss can be effectively treated with glucocorticoids. Patients to whom our therapy was offered after failure of primary and secondary glucocorticoid therapy might have been a selection of “glucocorticoid non- or poor-responders.” Lastly, a round window rupture or fistula as a cause of sudden hearing loss was only excluded visually. It cannot not be excluded, however, that patients with a non-visible fistula were part of the cohort, since objective measures, like Cochlin-tomoprotein (Ikezono et al., 2010) in middle ear lavage samples were not available in our department.

## Conclusion

Based on the observation in this case series, the extracochlear application of a PLGA-based biodegradable drug delivery system for the controlled release of dexamethasone in the RW niche of the human inner ear appears feasible and safe. Due to the uncontrolled study design and the tertiary therapy setting after failure of primary systemic and secondary intratympanic glucocorticoid therapy with a rather long period between ISSHL and start of this tertiary treatment, conclusions about the efficacy are limited. First observations on intracochlear application of the drug delivery system also demonstrated feasibility but did not show beneficial audiological outcome. However, since to date, clinical data on the use of biodegradable drug delivery systems for local inner ear therapy in humans is very limited, these findings are of importance for the development of such systems. Our observations may encourage the use of biodegradable implants as controlled release drug delivery systems earlier after ISSHL or in the therapy of inner ear disorders in general in the context of prospective controlled clinical trials.

## Data availability statement

The raw data supporting the conclusions of this article will be made available by the authors, without undue reservation on reasonable request.

## Ethics statement

The studies involving human participants were reviewed and approved by the Ethics Committee of the Martin Luther University. The patients/participants provided their written informed consent to participate in this study.

## Author contributions

SP and TR contributed to the conception and design of the study and organized the database. SP performed the surgical procedure and wrote the first draft of the manuscript. TR performed the statistical analysis of the audiological measurements. DB performed the histological analysis. AL and EL run the simulations. SP, AL, EL, DB, KM, and TR wrote the sections of the manuscript. All authors contributed to manuscript revision, read, and approved the submitted version.

## Footnotes

1
https://alecsalt.com

## Supplementary material

Supplementary Video material The video illustrates examples for the implantation of a solid biodegradable drug delivery system into the round window (RW) niche (part 1) and into scala tympani (part 2). **Part 1:** After raising a tympanomeatal flap, the middle ear and especially the oval and RW niches were inspected. The bony rim of the RW niche, i.e., the tegmen, the anterior postis and partially also the posterior postis were drilled away to adequately inspect the entire RW membrane. The implant was transferred into the RW with a 0.7 mm suction tip. The implant pieces were than covered with soft tissue. This was followed by application of fibrin glue. The tympanomeatal flap was then replaced, and a standard ear canal dressing was inserted. **Part 2:** After drilling the bony rim of the RW niche and placing the implant into the RW niche pointing toward the RW membrane a slit-like opening was made in the RW membrane using an otologic 90°hook. This instrument was also used to gently push the rod-shaped implant into the base of scala tympani. The RW was sealed with soft tissue and fibrin glue.
